# On finding minimal absent words

**DOI:** 10.1186/1471-2105-10-137

**Published:** 2009-05-08

**Authors:** Armando J Pinho, Paulo JSG Ferreira, Sara P Garcia, João MOS Rodrigues

**Affiliations:** 1 Signal Processing Lab, DETI/IEETA, University of Aveiro, 3810-193 Aveiro, Portugal

## Abstract

**Background:**

The problem of finding the shortest absent words in DNA data has been recently addressed, and algorithms for its solution have been described. It has been noted that longer absent words might also be of interest, but the existing algorithms only provide generic absent words by trivially extending the shortest ones.

**Results:**

We show how absent words relate to the repetitions and structure of the data, and define a new and larger class of absent words, called minimal absent words, that still captures the essential properties of the shortest absent words introduced in recent works. The words of this new class are minimal in the sense that if their leftmost or rightmost character is removed, then the resulting word is no longer an absent word. We describe an algorithm for generating minimal absent words that, in practice, runs in approximately linear time. An implementation of this algorithm is publicly available at .

**Conclusion:**

Because the set of minimal absent words that we propose is much larger than the set of the shortest absent words, it is potentially more useful for applications that require a richer variety of absent words. Nevertheless, the number of minimal absent words is still manageable since it grows at most linearly with the string size, unlike generic absent words that grow exponentially. Both the algorithm and the concepts upon which it depends shed additional light on the structure of absent words and complement the existing studies on the topic.

## Background

There has been recent interest in absent words in DNA sequences, which are words that do not occur in a given genome. At the individual level, such words can be used as biomarkers for potential preventive and curative medical applications as derived from personal genomics efforts, while at the group level the comparison of genetic traits may impact, for example, on population genetics, or evolutionary profiles obtained from comparative genomics. It is therefore not surprising that absent words have been the subject of recent studies [1-3].

Hampikian and Andersen [1] used the term "*nullomer*" to designate the shortest words that do not occur in a given genome and the term "*prime*" to refer to the shortest words that are absent from the entire known genetic data. Herold *et al*. [3] used the term "*unword*" also to designate the shortest absent words. According to the definition, any given DNA sequence has nullomers/unwords of a certain size, that are uniquely defined for that sequence, and also of the shortest possible size.

The algorithm used by Hampikian and Andersen [1] to obtain the absent words tracks the occurrence of all possible words up to a user-specified length limit *n*, using a set of 4^*n *^counters for the 4^*n *^possible words of length *n*. This yields the existing absent words up to the given length limit, *n*. The approach taken by Herold *et al*. [3] has some computational advantages over that of Hampikian and Andersen [1], by being less demanding in terms of memory needs and processing time.

In this paper, we generalize the concept of nullomer/unword, such that other words, not necessarily the shortest ones, can be included (for a precise definition see Definition 3). In fact, the original definition adopted by Hampikian and Andersen [1] and by Herold *et al*. [3] might be too limiting, because there are sequences that have only a few nullomers/unwords. For example, and according to the results presented in [3], the genome of the worm, *Caenorhabditis elegans*, has two nullomers/unwords, whereas the genome of the extreme thermophile, *Thermococcus kodakarensis*, has only one.

As stated by Herold *et al*. [3], longer absent words may also be of interest. For generating those longer absent words, they propose adding all unwords (say, of size *k*) as additional sequences to the genome and re-running the program. These additional absent words, which we call generic absent words and denote by , also include extended nullomers/unwords, i.e., words that contain nullomers/unwords. However, not all generic absent words are trivial extensions of nullomers/unwords.

Nullomers/unwords satisfy the following property, *P*.

**Property 1 (*P*)**. *If the leftmost or the rightmost character of a given nullomer/unword is removed, then the resulting word is no longer an absent word*.

This property *P *does not hold for the absent words obtained by trivially extending nullomers/unwords nor for the longer absent words suggested by Herold *et al*. [3]. In other words, for a generic absent word, there is no way of knowing in advance if the elimination of some characters from one of the extremities of the word yields an absent word or not.

**Figure 1:**
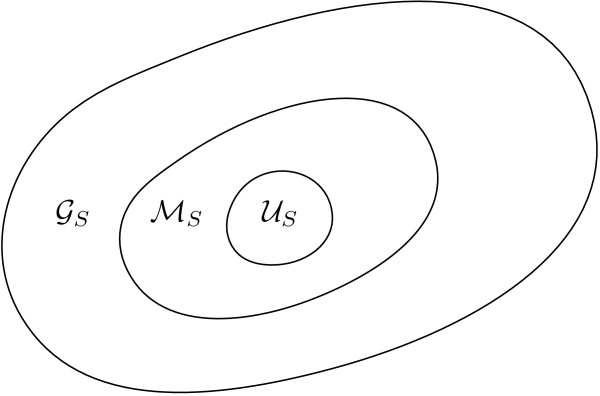
**Relation between the sets of nullomers/unwords, , minimal absent words, ℳ_*S*_, and generic absent words, , of a given string *S***.

These observations motivated this paper, leading us to the definition of what we call minimal absent words, denoted by ℳ_*S*_, which are absent words (although not necessarily the shortest ones) for which property *P *holds. Figure 1 presents a diagram showing the relation between the generic absent words, the minimal absent words proposed in this paper, and the nullomers/unwords (denoted by ). Note that, as the size *n *of the word grows, the number of generic absent words of size *n *approaches 4^*n*^. On the contrary, as we will show later, the total number of minimal absent words of a string *S *is upper bounded by |*S*||Σ|^2^, where |*S*| denotes the size of the string and |Σ| is the alphabet size.

We have developed an efficient algorithm for computing these minimal absent words, which, in practice, runs in approximately linear time. Our work can be regarded as a complement to the works of Hampikian and Andersen [1] and of Herold *et al*. [3], in the sense that it provides a generalization of the nullomer/unword concept previously introduced, and helps to clarify their structure.

## Methods

### Basic definitions

Let *S *be a string over a finite alphabet Σ. We denote by *S*[*p*], 1 ≤ *p *≤ |*S*| the *p*th character of *S*, where |*S*| designates the length (i.e., number of characters) of *S*, and by *S*[*p*_1_..*p*_2_], *p*_1 _≤ *p*_2 _the substring of *S *that starts at position *p*_1 _and ends at position *p*_2_. Therefore, *S*[1..*p*] denotes a prefix of *S *and *S*[*p*..|*S*|] a suffix. *Sr *indicates the concatenation of character *r *to the right end side of string *S*, whereas *lS *indicates the concatenation of character *l *to the left end side of *S*.

For convenience, we define two additional virtual characters, # and $. They are virtual in the sense that they do not belong to the alphabet Σ. By definition, the character to the left of the first character of the string is #, and the character to the right of the last character of the string is $. In other words, we define *S*[0] = # and *S*[|*S*| + 1] = $.

Let , where *α *is a substring of *S*, to be the set of positions of *S *where *α *occurs, so that *S*[*p*..*p *+ |*α*| - 1] = *α*, ∀*p *∈  and *S*[*p*..*p *+ |*α*| - 1] ≠ *α*, ∀*p *∉ . We define  and  to be the sets of characters that appear, respectively, to the immediate left and right of the several occurrences of *α*, and the sets  and . We also denote by ℰ_*α *_⊆ Σ × Σ the set of all pairs of characters (*S*[*p *- 1], *S*[*p *+ |*α*|]), ∀*p *∈ , i.e., all pairs of characters "enclosing" the occurrences of *α*.

**Definition 1 (Maximal repeated pair **[4]). *A maximal repeated pair in a string S is a triple *(*p*_1_, *p*_2_, *α*), *such that p*_1 _≠ *p*_2_, *p*_1_, *p*_2 _∈ , *S*[*p*_1 _- 1] ≠ *S*[*p*_2 _- 1] *and S*[*p*_1 _+ |*α*|] ≠ *S*[*p*_2 _+ |*α*|].

**Definition 2 (Maximal repeat **[4]). *A substring α is a maximal repeat of S if there is at least a maximal repeated pair in S of the form *(*p*_1_, *p*_2_, *α*).

### Characterization

We are now ready to formally introduce the concept of minimal absent word.

**Definition 3 (Minimal absent word)**. *A string γ*, |*γ*| ≥ 3, *is a minimal absent word of S if γ is not a substring of S, but γ*[2..|*γ*|] *and γ*[1..|*γ*| - 1] *are substrings of S*.

**Example 1**. *Consider the following example, where *Σ = {*A*, *C*, *G*, *T*} *and S *= *ACTAACTG. According to Definition 3, the set of minimal absent words of S is*



Note that the set of nullomer/unwords (of size at least three) is



*and that the set of generic absent words, , is too large to be of any practical interest*.

**Theorem 1**. *If lαr is a minimal absent word of string S, then α is a maximal repeat in S*.

*Proof*. According to Definition 3, if *lαr *is a minimal absent word of *S*, then *lα *and *αr *are substrings of *S*, i.e., *lα *= *S*[*p*_1_..*p*_1 _+ |*α*|] and *αr *= *S*[*p*_2_..*p*_2 _+ |*α*|], with *p*_2 _≠ *p*_1 _+ 1 (if *p*_2 _= *p*_1 _+ 1 then *lαr *would be a substring of *S*, contradicting the assumption that it is a minimal absent word). Now consider that the character to the immediate right of *lα *is *r' *= *S*[*p*_1 _+ |*α*| + 1] and that the character to the immediate left of *αr *is *l' *= *S*[*p*_2 _- 1]. Because *lαr *does not exist in *S*, then *l' *cannot be the same character as *l *and *r' *cannot be the same character as *r*, implying that (*p*_1 _+ 1, *p*_2_, *α*) is a maximal repeated pair and, therefore, *α *is a maximal repeat in *S*.   □

Note that this applies to minimal absent words with at least three characters, according to Definition 3. The restriction could be removed by allowing *α *to be the empty string. For the sake of clarity, we do not consider this case here. In fact, directly finding minimal absent words of length two requires |Σ|^2 ^string matching operations, which can be performed in a reasonable time, unless the size of the alphabet is unusually large. This is why in Definition 3 we restricted the size of a minimal absent word to be at least three.

**Theorem 2**. *A string lαr is a minimal absent word of S if and only if *(*l*, *r*) ∉ ℰ_*α*_, *for l *∈ ℒ_*α *_*and r *∈ ℛ_*α*_.

*Proof*. If (*l, r*) ∉ ℰ_*α*_, then in none of the occurrences of *α *in *S *we have, simultaneously, a *l *character to the immediate left of *α *and a *r *character to its immediate right, implying that *lαr *does not occur in *S*. On the other hand, since *l *∈ ℒ_*α*_, then there is at least one position in *S *where the substring *lα *occurs, the same holding for the *αr *substring, because *l *∈ ℛ_*α*_. Therefore, according to Definition 3, *lαr *is a minimal absent word of *S*.

Now, consider that *lαr *is a minimal absent word of *S *and (*l*, *r*) ∈ ℰ_*α*_. In that case, there would be a substring *lαr *in *S*, contradicting the assumption that *lαr *is a minimal absent word.   □

### Finding the minimal absent words

Theorem 1 states that all minimal absent words are associated with maximal repeats. Therefore, finding all minimal absent words may be associated to finding all maximal repeats in a string, which can be done using suffix trees in *O*(|*S*|) time [4]. Moreover, suffix trees can be built and stored also in *O*(|*S*|) time/memory, respectively [5-7]. See [4] for an introduction to suffix trees.

#### Suffix trees

A suffix tree of a string *S *is a rooted directed tree with exactly |*S*| leaves (numbered 1 to |*S*|). Each internal node, other than the root, has at least two children and each edge is labeled with a nonempty substring of *S*. No two edges out of a node can have edge-labels beginning with the same character. For any leaf *p*, the concatenation of the edge-labels on the path from the root to leaf *p *corresponds to the suffix that starts at position *p*, i.e., to *S*[*p*..|*S*|].

The condition stating that each internal node, other than the root, should have at least two children, implies that some strings do not have a suffix tree representation. In fact, this condition is violated in strings having a suffix that is a prefix of another suffix. To remedy this, a character that does not appear in any other position of *S *is usually appended at the end of the string (the "$" character is frequently used for this purpose [4]).

**Figure 2:**
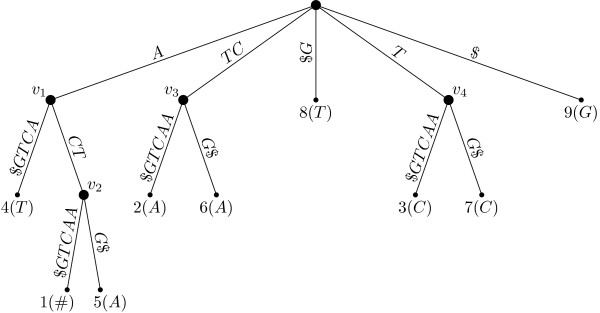
**Suffix tree for string *S*$ = *ACT AACT G*$**.

Figure 2 shows the suffix tree corresponding to the string of Example 1, using "$" as the terminating character.

**Definition 4 (Left character **[4]). *For each position p in S, character S*[*p *- 1] *is called the left character of p. The left character of a leaf of the suffix tree is the left character of the suffix represented by that leaf*.

The characters appearing inside parentheses near the leaves of the suffix tree of Fig. 2 are the corresponding left characters. Notice the # character associated with leaf number one, corresponding to *S*[0].

**Definition 5 (Left diverse **[4]). *A node v of the suffix tree is called left diverse if at least two leaves in v's subtree have different left characters*.

In the suffix tree depicted in Fig. 2, nodes *v*_1 _and *v*_2 _are the only left diverse nodes. According to Theorem 7.12.2 of [4], the string *α *labeling the path to a node *v *of the suffix tree is a maximal repeat if and only if *v *is left diverse. Therefore, in Fig. 2, the substrings formed along the paths from the root node to each of the two nodes *v*_1 _and *v*_2 _correspond to maximal repeats. Those strings are *A *and *ACT*, which are the base of the minimal absent words *AAA*, *TAC *and *AACTA*. Recall that, in a string *S*, there might be, at most, |*S*| maximal repeats (Theorem 7.12.1 in [4]). This implies that the number of minimal absent words of a string *S *is upper bounded by |*S*||Σ|^2^.

#### Suffix arrays

Suffix trees are a powerful data structure that allowed important advances in string processing [4]. However, the space required by a suffix tree, although growing linearly with the size of the string, might still be excessive for some applications [8,9].

Suffix arrays are an alternative data structure that is more space efficient (4 bytes per input character for strings of size up to 2^32^, in its basic form). However, to increase the efficiency of certain tasks, they might require auxiliary information [9]. Introduced in [10,11], the suffix arrays can be constructed in linear time from the corresponding suffix tree [4] or using direct algorithms [12-14].

Basically, a suffix array is an array of integers, *p*_*k*_, 1 ≤ *p*_*k *_≤ |*S*|, 1 ≤ *k *≤ |*S*|, each pointing to the beginning of a suffix of *S*, such that *S*[*p*_*i*_..|*S*|] lexicographically precedes than *S*[*p*_*j*_..|*S*|], ∀*i *<*j*. Table 1 shows the suffix array of string *S *= *ACTAACTG *(*p*_*k *_column), as well as two auxiliary arrays: the longest common prefix array, lcp-array, and the left character array, bwt-array, which corresponds to the Burrows and Wheeler transform [15].

**Table 1:** Suffix array *p*_*k *_and auxiliary information, in this case the lcp and bwt arrays, for *S *= *ACTAACTG*.

*k*	*p*_*k*_	*lcp*_*k*_	*bwt*_*k*_	*S*[*p*_*k*_..|*S*|]
1	4	0	*T*	*AACTG*
2	1	1	#	*ACTAACTG*
3	5	3	*A*	*ACTG*
4	2	0	*A*	*CTAACTG*
5	6	2	*A*	*CTG*
6	8	0	*T*	*G*
7	3	0	*C*	*TAACTG*
8	7	1	*C*	*TG*

**Table 2:** Generalized suffix array *p*_*k *_and auxiliary information for strings *S*_1 _= *ACTAACTG *and *S*_2 _= *CGTACTA*.

*k*	*p*_*k*_	*lcp*_*k*_	*bwt*_*k*_	*S*[*p*_*k*_..|*S*|]
1	16	0	*T*	*A*
2	4	1	*T*	*AACTG*
3	13	1	*T*	*ACTA*
4	1	4	#	*ACTAACTG*
5	5	3	*A*	*ACTG*
6	10	0	#	*CGTACTA*
7	14	1	*A*	*CTA*
8	2	3	*A*	*CTAACTG*
9	6	2	*A*	*CTG*
10	8	0	*T*	*G*
11	11	1	*C*	*GTACTA*
12	15	0	*C*	*TA*
13	3	2	*C*	*TAACTG*
14	12	2	*G*	*TACTA*
15	7	1	*C*	*TG*

The lcp-array contains the lengths of the longest common prefix between consecutive ordered suffixes, i.e., *lcp*_*k *_indicates the length of the longest common prefix between *S*[*p*_*k*-1_..|*S*|] and *S*[*p*_*k*_..|*S*|], 2 ≤ *k *≤ |*S*|. By convention, *lcp*_1 _= *lcp*_|*S*|+1 _= 0.

The bwt-array is a permutation of *S*, such that *bwt*_*k *_= *S*[*p*_*k *_- 1]. Remember that the character to the immediate left of *S*[1] has been defined to be #, a convention that explains the value of *bwt*_*k *_for *p*_*k *_= 1. Conceptually, the bwt-array does not provide additional information, because the left character of any character of *S *can be determined by direct access to *S*. In fact, in this paper, we use both notations, *bwt*_*k *_and *S*[*p*_*k *_- 1], interchangeably. However, in practice, the bwt-array allows sequential memory access and hence improves the performance, due to better cache use [16].

**Definition 6 (Lcp-interval)**. *Interval *[*i*..*j*], 1 ≤ *i *<*j *≤ |*S*|, *is an lcp-interval of lcp-depth d, denoted *⟨*d*, *i*, *j*⟩, *if*

*1. lcp*_*i *_<*d*,

*2. lcp*_*k *_≥ *d*, ∀*i *<*k *≤ *j*,

*3. lcp*_*k *_= *d*, *for at least one k in i *<*k *≤ *j*,

*4. lcp*_*j*+1 _<*d*.

The lcp-intervals of the example string *S *= *ACTAACTG *are ⟨1, 1, 3⟩ and ⟨1, 7, 8⟩ of lcp-depth 1, ⟨2, 4, 5⟩ of lcp-depth 2, and ⟨3, 2, 3⟩ of lcp-depth 3. Note that each of these lcp-intervals correspond to a distinct internal node of the suffix tree (see Fig. 2). For example, the lcp-interval ⟨1, 1, 3⟩ is associated with node *v*_1_, whereas the lcp-interval ⟨3, 2, 3⟩ corresponds to node *v*_2_. Therefore, we can think of a virtual tree of lcp-intervals having a structure similar to the corresponding suffix tree [16].

This correspondence between lcp-intervals and internal nodes of the suffix tree is important, because it helps mapping some concepts from the suffix tree data structure into the suffix array approach. For example, the notion of left diverse node can be mapped directly into the lcp-intervals. Finding if a node, associated with lcp-interval ⟨*d*, *i*, *j*⟩, is left diverse, is the same as finding if at least two characters of *bwt*_*k *_differ, for *i *≤ *k *≤ *j*. Moreover, in that case, the corresponding maximal repeat is, for example, *α *= *S*[*p*_*i*_..*p*_*i *_+ *d *- 1] (note that all substrings *S*[*p*_*k*_..*p*_*k *_+ *d *- 1], ∀*i *≤ *k *≤ *j*, are identical).

Algorithm 1 (adapted from [16,17]) generates all lcp-intervals using the lcp-array and a stack. The "Push" and "Pop" operations have the usual meaning when associated to stack processing. The variable "*top*" refers to the lcp-interval, ⟨*d*, *i*, *j*⟩, on the top of the stack.

**Algorithm 1**. *Computation of lcp-intervals*.

   Push ⟨0, 0, 0⟩

   **for ***k *= 2 to |*S*| **do**

      *i *← *k *- 1

      **while ***lcp*_*k*_<*top.d ***do**

         *lcpint *← Pop

         *lcpint.j *← *k *- 1

         Process *lcpint*

         *i *← *lcpint.i*

      **end while**

      **if ***lcp*_*k *_> *top.d ***then**

         Push ⟨*lcp*_*k*_, *i*, 0⟩

      **end if**

   **end for**

In order to find the minimal absent words, the function "Process" in Algorithm 1 executes Algorithm 2, that builds the ℒ_*α*_, ℛ_*α *_and ℰ_*α *_sets, determines if the lcp-interval is left diverse, and, if true, outputs the minimal absent words associated with the lcp-interval ⟨*d*, *i*, *j*⟩.

**Algorithm 2**. *Computation of the minimal absent words for a given lcp-interval *⟨*d*, *i*, *j*⟩, *where α *= *S*[*p*_*i*_..*p*_*i *_+ *d *- 1].

    ← ∅

    ← ∅

   ℰ_*α *_← ∅

   **for ***k *= *i *to *j ***do**

       ←  ∪ {*S*[*p*_*k *_- 1]}

       ←  ∪ {*S*[*p*_*k *_+ *d*]}

      **if ***S*[*p*_*k *_- 1] ≠ # and *S*[*p*_*k *_+ *d*] ≠ $ **then**

         ℰ_*α *_← ℰ_*α *_∪ {(*S*[*p*_*k *_- 1], *S*[*p*_*k *_+ *d*])}

      **end if**

   **end for**

   **if **|| > 1 **then **{Left diverse}

      **for all ***l *∈ ℒ_*α *_**do**

         **for all ***r *∈ ℛ_*α *_**do**

            **if **(*l*, *r*) ∉ ℰ_*α *_**then**

               Substring *lαr *is a minimal absent word

            **end if**

         **end for**

      **end for**

   **end if**

Our results remain valid for sets of strings  = {*S*_1_, *S*_2_,..., *S*_*z*_} over a finite alphabet Σ. In this case, the minimal absent words are generated through the concatenation of the strings using a delimiting character not belonging to the alphabet. The delimiter avoids the creation of artificial substrings across string boundaries.

For example, with  = {*S*_1_, *S*_2_}, where



and



the set of minimal absent words of  is



whereas the sets of minimal absent words for each string are



and



Table 2 shows the (generalized) suffix array [18] associated to strings *S*_1 _and *S*_2_.

## Results and discusion

**Figure 3:**
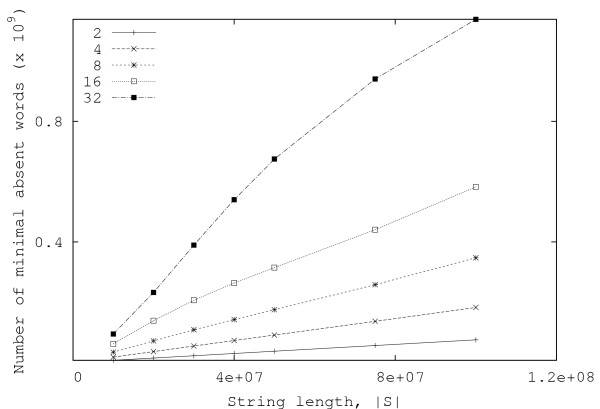
**Number of minimal absent words for random strings**. Plots of the number of minimal absent words as a function of the string length, for random strings with |Σ| = 2, 4, 8, 16, 32.

**Figure 4:**
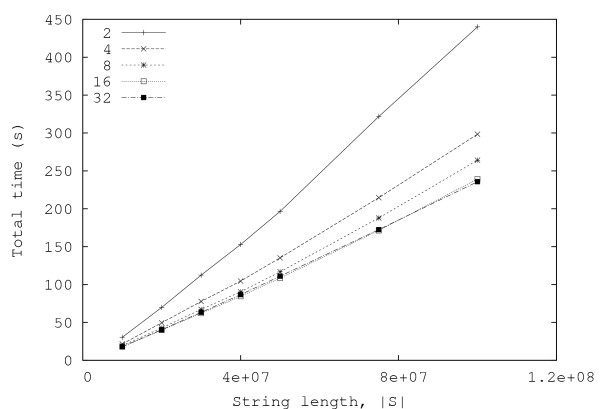
**Total time for generating all minimal absent words for random strings**. Plots of the total time required for generating all minimal absent words (including the time needed for reading the data and creating the suffix and lcp arrays), as a function of the string length, for random strings with alphabet size |Σ| = 2, 4,8, 16, 32.

In this section, we present some experimental results obtained both with random and real data. Figures 3 and 4 show, respectively, the total number of minimal absent words, |ℳ_*S*_|, and the total time required for computing them, for random strings over alphabets with |Σ| = 2, 4, 8, 16, 32. Each point in the graphics is the average of ten independent runs. These results have been obtained with an Intel Core 2 Duo laptop computer (clocked at 1.66 GHz and with 2 GByte of RAM).

The graphic displayed in Fig. 4 shows an apparently curious behavior: the time taken by the algorithm increases as the size of the alphabet decreases. This might be due to the fact that, for the same string length, strings over smaller alphabets imply deeper suffix trees and, since the lcp-intervals are related to the internal nodes of the suffix tree, generating them for smaller alphabets takes longer.

From the curves presented in Fig. 4, it can be seen that, in practice, the running time of the algorithm is approximately linear with the length of the string. Moreover, the number of minimal absent words (displayed in Fig. 3) also shows a similar behavior, contrasting with the exponential growth of the number of generic absent words of a string.

**Figure 5:**
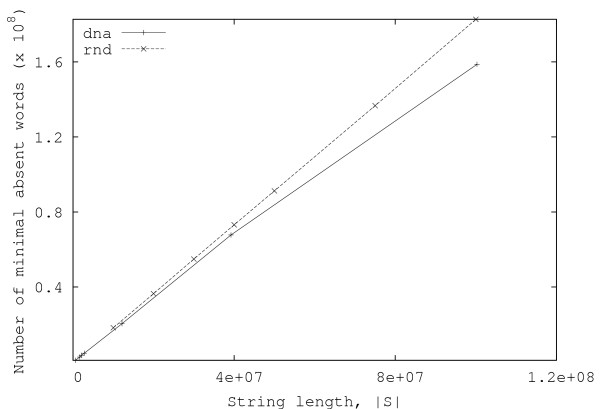
**Number of minimal absent words for several nucleotide sequences**. Plots of the number of minimal absent words, as a function of the string length, for several nucleotide sequences ("dna" curve). The number of minimal absent words for random strings with alphabet size |Σ| = 4 is also included for comparison ("rnd" curve).

**Figure 6:**
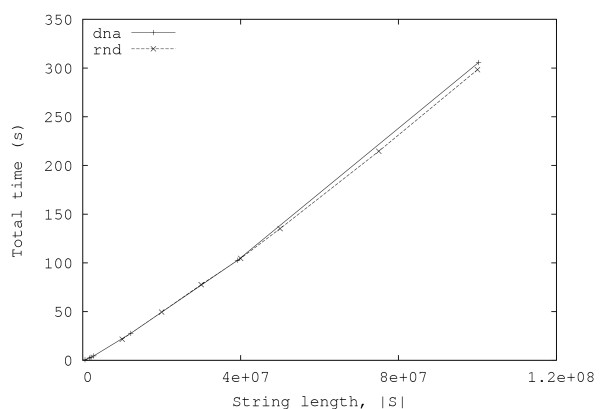
**Total time for generating all minimal absent words for several nucleotide sequences**. Plots of the total time required for generating all minimal absent words (including the time needed for reading the data and creating the suffix and lcp arrays), as a function of the string length, for several nucleotide sequences ("dna" curve). The total time required for generating all minimal absent words for random strings with alphabet size |Σ| = 4 is also included for comparison ("rnd" curve).

In Table 3, we present the number of minimal absent words and the number of generic absent words, i.e., all absent words, even those composed of shorter absent words, for the same organisms used in [3]. We have adopted the notation  and  for designating, respectively, the number of minimal absent words and the number of generic absent words of length *n *associated with string *S*. Our method provided the same number of nullomers/unwords (which coincides with the number of smallest minimal absent words) reported in [3], except for the budding yeast, *Saccharomyces cerevisiae*, (two, instead of the reported four) and the mouse, *Mus musculus*, (190 instead of 192). Nevertheless, the software provided by Herold *et al*. [3] reported two unwords for the *S. cerevisiae *and 190 for the *M. musculus *data that we used. Figures 5 and 6 show the total number of minimal absent words and the running time for some of the DNA sequences mentioned in Table 3. For comparison, those figures also include the results obtained with random strings over an alphabet of size four. As can be seen, the total number of minimal absent words obtained with real DNA sequences is slightly less than the number obtained with random strings, whereas the time required for producing the minimal absent words is roughly identical to the time required when using random data.

**Table 3:** Number of minimal absent words and generic absent words for some genomes.

Organism	Reference	Genome size			Length, *n*
			104	104	11
*H. sapiens*	Release 36.1	≈ 2.9 Gb	44 149	44 970	12
			2 039 862	2 368 682	13

			190	190	11
*M. musculus*	Release m36.1	≈ 2.6 Gb	52 087	53 573	12
			2 192 708	2 579 838	13

			104	104	11
*D. melanogaster*	FB 5	≈ 162 Mb	172 849	173 674	12
			10 040 282	11 335 034	13

			2	2	10
*C. elegans*	WB 170	≈ 100 Mb	7 664	7 680	11
			1 092 286	1 151 728	12

			2 262	2 262	11
*N. crassa*	Assembly 7	≈ 39 Mb	1 064 938	1 082 787	12
			20 213 298	27 903 272	13

			2	2	9
*S. cerevisiae *S228C	SGD 1	≈ 12 Mb	6 435	6 450	10
			414 520	462 882	11

			248	248	8
*S. aureus *MSSA476	NC002953	≈ 2.8 Mb	11 908	13 744	9
			162 113	251 497	10

			1	1	8
*T. kodakarensis*	NC006624	≈ 2.09 Mb	2 314	2 322	9
			136 917	154 340	10

			3	3	6
*M. jannaschii*	NC000909	≈ 1.66 Mb	126	150	7
			3 790	4 834	8

			5	5	6
*M. genitalium*	NC000908	≈ 0.58 Mb	340	380	7
			6 156	8 733	8

The notation  corresponds to the number of minimal absent words of length n associated with string S, whereas  has a similar meaning but for the case of generic absent words. The generic absent words have been generated using publicly available software provided by Herold et al. 3. The organisms are sorted according to decreasing genome size, which refers to the number of unambiguous bases of the genome. The reversed complement of the sequences has been considered in the generation of the results.

**Figure 7:**
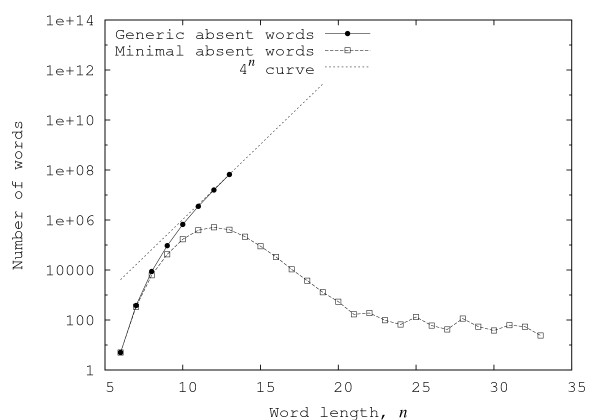
**Example of the growth of the number of generic absent words and minimal absent words as a function of word size, *n***. Plots of the number of generic absent words and minimal absent words for the case of the *M. genitalium *organism. It can be seen that the number of minimal absent words grows until a maximum and then decreases towards zero. On the contrary, the number of generic absent words grows exponentially. For comparison, we also included the graphic of the function 4^*n*^. This behavior has also been observed in the other sequences.

Figure 7 shows how the number of generic absent words and minimal absent words grow as a function of the length of the word, *n*. As can be observed, the number of minimal absent words grows until a maximum value and then decreases beyond that point. In opposition, the number of generic absent words grows exponentially. This is confirmed by the 4^*n *^curve also plotted in Fig. 7.

## Conclusion

Words absent from DNA data have been the subject of recent studies [1-3]. In this paper, we provided a precise characterization of a class of absent words, named minimal absent words, that extends the class previously discussed of nullomers/unwords. Our minimal absent words share with nullomers/unwords the property of being minimal, that is, the removal of one character from either end of a nullomer/unword yields an existing word. The set of minimal absent words is much larger than the set of nullomers/unwords, and, therefore, potentially more useful for applications that require a richer variety of absent words. We also proposed an algorithm for generating the minimal absent words that is based on suffix arrays and that, in practice, runs in approximately linear time. We hope that this algorithm and the concept of minimal absent word may shed some more light on the structure of absent words and complement the existing studies on the topic.

## Authors' contributions

The algorithms have been developed by AP, PF and JR, and implemented in the C programming language under the Linux operating system by AP and PF. Testing was performed by AP and SG. All authors have contributed to the writing and improvement of the final manuscript.
